# Renoprotective effects of garlic

**DOI:** 10.12861/jrip.2013.09

**Published:** 2013-03-01

**Authors:** Hamid Nasri

**Affiliations:** ^1^Department of Nephrology, Division of Nephropathology, Isfahan University of Medical Sciences, Isfahan, Iran

**Keywords:** Renoprotection, Garlic extract, Diabetic nephropathy

**Implication for health policy/practice/research/medical education:**

Combination of metformin and garlic extract may ameliorate diabetic nephropathy.

Recently an article published, entitled “renoprotective effect of aged garlic extract in streptozotocin-induced diabetic rats” (1). In this study, diabetic rats were feed by aged garlic juice orally. The serum and urinary biochemical parameters were analyzed in all the groups and at the end of 12 weeks follow up, the kidney morphological examination were performed, too. They found that, diabetic rats had a significant change in the urine and serum constituents such as creatinine, urea, albumin and glycated hemoglobin. Additionally, the serum lipid profile of the diabetic rats was altered significantly compared to that of the control rats. However, the diabetic rats, which supplemented with aged garlic juice, restored all these biochemical changes. The efficiency of the aged garlic juice was substantiated by the morphological changes in the kidney, too. They concluded that aged garlic juice has the ability to ameliorate kidney injury in diabetic rats and the renoprotective effect of aged garlic juice may be attributed to its anti-glycation and hypolipidemic activities.We would like to remind a few points about this study. Recently, to find the ameliorative properties of metformin on kidney biochemical and histologic alterations of gentamicin-induced kidney damage in rats, we conducted a preclinical investigation (2).In this study, we found attenuation of gentamicin-induced acute kidney injury in rats. More recently, to test the efficacy of co-administration of garlic extract and metformin for prevention of gentamicin–renal tubular injury, we conducted another study on 70 male Wistar rats (3). The results of this study demonstrate that metformin and garlic juice or their combination has both curative and protective effects against gentamicin nephrotoxicity (3). Diabetic kidney disease is one of the most important complications of diabetes mellitus (4),and metformin has been broadly used for treatment of type 2 diabetes (4,5). Thus the study of Shiju *et al*. further attests our results and those published by previous investigators which garlic extract protects against tubular injury by restoring the biochemical alterations and modulation of oxidative stress on the tubules (3-5). Furthermore, in diabetic nephropathy, there is also tubular cell injury due to glycosuria (5). These findings can more potentiate the clinical use of combination of metformin and garlic extract in the prevention of diabetic nephropathy. In this regard, to better understand the garlic kidney protective properties, more animal and clinical studies are suggested.

## Author’s contribution

HN is the single author of the paper.

## Conflict of interests

The author declared no competing interests.

## Ethical considerations

Ethical issues (including plagiarism, data fabrication, double publication) have been completely observed by the author.

## Funding/Support

None.
